# Supplementary material for: Applicability and potential of monitored reconstruction in computed tomography
Source: PLoS One. 2024 Jul 22;19(7):e0307231. doi: 10.1371/journal.pone.0307231 (PMC11262673; doi:10.1371/journal.pone.0307231)
Supplement: Attachment — Submitted filename: Plos one_comments.pdf [file pone.0307231.s001.pdf]

Applicability and potential of monitored reconstruction in computed tomography

The article represents the empirical quantitative model for calculating the acquisition and reconstruction times of MTR. It is demonstrated that projection acquisition order has a significant impact on the time and dose of tomographic experiments. To estimate the restrictions and scope of applicability for MTR four typical commercial setups are studied within a proposed model.

Comments

1. Abstract seems general, please mention the specific techniques and optimization methods the author adopted for the experiment. Also, quantify the proposed method values with existing methods in the abstract.
2. Figures are not embedded in the article. It is difficult to review the manuscript.
3. What is FDK? Author used two reconstruction algorithms such as SIRT, and FBP. This is compared with different CT cases. Kindly explain more about, what the improvements are done by authors in the proposed algorithm compared with typical methods.
4. The author has to include the algorithm and flow chart of the algorithm in the article.
5. Author mentioned the experiments and demonstration setups for the reconstructions. Those are missing in the article.
6. Please quantify the reconstruction parameters such as acquisition time with existing methods. It is helpful to understand the processing time of the scanning.
7. What are the other parameters to measure the quality of the reconstruction?
8. What are software and hardware used for the method?
